# Supplementary material for: Global organisation of structural covariance networks derived from parcellated cortical surface area in atypical populations
Source: bioRxiv. 2025 Oct 30:2025.10.29.685428. Preprint. [Version 2] doi: 10.1101/2025.10.29.685428 (PMC12636564; doi:10.1101/2025.10.29.685428)
Supplement: Supplement 1 [file media-1.pdf]

1 # Supplementary Material

Sample	Clusters normal (%)	Clusters different variance (%)
Syn100	Control: 85.48<br>Aff: 72.58	0.00
ABIDE-I	Control: 74.19<br>Aff: 85.48	0.00
ABIDE-II	Control: 70.97<br>Aff: 59.68	8.33
HCP-EP	Control: 85.48<br>Aff: 79.03	1.11
BANDA	Control: 79.03<br>Aff: 87.1	0.00
DCAM	Control: 83.87<br>Aff: 61.29	2.78
PDC	Control: 90.32<br>Aff: 79.03	0.00

Table S1: Proportions of clusters passing tests for normality and group differences in variances. Anderson-Darling test for normality and F-test for variance difference, $P < .05$ with FDR correction, $N = 102$.

Cluster number	F-value	F-test P-value	Clustering	Global efficiency	Path length
65	0.74	$<.001$	Control: 0.137<br>Syn: 0.158	Control: 0.177<br>Syn: 0.193	Control: 3.11<br>Syn: 2.87
64	0.76	$<.001$	Control: 0.137<br>Syn: 0.159	Control: 0.177<br>Syn: 0.193	Control: 3.10<br>Syn: 2.86
63	0.76	$<.001$	Control: 0.137<br>Syn: 0.158	Control: 0.177<br>Syn: 0.195	Control: 3.11<br>Syn: 2.84
61	0.81	$<.001$	Control: 0.138<br>Syn: 0.164	Control: 0.178<br>Syn: 0.201	Control: 3.09<br>Syn: 2.75
60	0.82	$<.001$	Control: 0.137<br>Syn: 0.164	Control: 0.179<br>Syn: 0.201	Control: 3.07<br>Syn: 2.76
59	0.83	$<.001$	Control: 0.135<br>Syn: 0.164	Control: 0.177<br>Syn: 0.201	Control: 3.11<br>Syn: 2.75

Table S2: Group differences in partial correlation variance and network metrics across different numbers of clusters in the Syn100 dataset. Different numbers of clusters were selected by varying the threshold for cutting the hierarhical clustering dendrogram around the optimal.

Sample	Clustering	Clustering effect size	Clustering P-value	Global efficiency	Global efficiency effect size	Global efficiency P-value	Path length	Path length effect size	Path length P-value
Syn100	Control: 0.037<br>Aff: 0.038	0.16	.4299	Control: 0.156<br>Aff: 0.172	1.53	.0631	Control: 3.539<br>Aff: 3.205	-1.58	.0605
ABIDE-I	Control: 0.038<br>Aff: 0.034	-1.34	.9183	Control: 0.170<br>Aff: 0.182	1.13	.1274	Control: 3.271<br>Aff: 3.062	-1.13	.1282
ABIDE-II	Control: 0.037<br>Aff: 0.036	-0.43	.6767	Control: 0.168<br>Aff: 0.183	1.41	.0771	Control: 3.323<br>Aff: 3.042	-1.56	.0592
HCP-EP	Control: 0.037<br>Aff: 0.036	-0.33	.3648	Control: 0.178<br>Aff: 0.171	-0.62	.2611	Control: 3.115<br>Aff: 3.254	0.76	.2215
BANDA	Control: 0.038<br>Aff: 0.040	0.73	.2205	Control: 0.158<br>Aff: 0.161	0.27	.3918	Control: 3.516<br>Aff: 3.434	-0.4	.3455
DCAM	Control: 0.036<br>Aff: 0.037	0.18	.5658	Control: 0.167<br>Aff: 0.170	0.33	.6332	Control: 3.320<br>Aff: 3.266	-0.27	.6067
PDC	Control: 0.038<br>Aff: 0.040	0.63	.7445	Control: 0.171<br>Aff: 0.171	0.04	.5129	Control: 3.235<br>Aff: 3.225	-0.04	.5155

Table S3: Group differences in global network metrics for positive-only SCNs. P-values were calculated via permutation tests with $n = 10,000$ permutations. Effect size for each measure is calculated using the observed group difference in metrics normalised by the null (shuffled) distribution standard deviation.

Sample	Clustering	Clustering effect size	Clustering P-value	Global efficiency	Global efficiency effect size	Global efficiency P-value	Path length	Path length effect size	Path length P-value
Syn100	Control: 0.092<br>Aff: 0.115	1.32	.0838	Control: 0.122<br>Aff: 0.138	1.83	**.0336**	Control: 4.680<br>Aff: 4.178	-1.85	**.0328**
ABIDE-I	Control: 0.087<br>Aff: 0.108	1.13	.1147	Control: 0.126<br>Aff: 0.138	1.24	.1057	Control: 4.475<br>Aff: 4.153	-1.28	.0995
ABIDE-II	Control: 0.089<br>Aff: 0.110	1.1	.1188	Control: 0.127<br>Aff: 0.138	1.2	.1099	Control: 4.421<br>Aff: 4.133	-1.17	.1189
HCP-EP	Control: 0.111<br>Aff: 0.101	-0.53	.2786	Control: 0.138<br>Aff: 0.129	-1.03	.1503	Control: 4.127<br>Aff: 4.426	1.24	.1088
BANDA	Control: 0.096<br>Aff: 0.096	-0.03	.5179	Control: 0.125<br>Aff: 0.129	0.43	.3341	Control: 4.559<br>Aff: 4.413	-0.56	.2918
DCAM	Control: 0.099<br>Aff: 0.102	0.13	.5640	Control: 0.129<br>Aff: 0.127	-0.2	.4215	Control: 4.412<br>Aff: 4.522	0.47	.3184
PDC	Control: 0.109<br>Aff: 0.112	0.16	.5695	Control: 0.135<br>Aff: 0.137	0.34	.6394	Control: 4.244<br>Aff: 4.180	-0.24	.5951

Table S4: Group differences in global network metrics for negative-only SCNs. P-values were calculated via permutation tests with $n = 10,000$ permutations. Effect size for each measure is calculated using the observed group difference in metrics normalised by the null (shuffled) distribution standard deviation.

Sample	Δ mean	Δ mean P-value	Δ variance	Δ variance P-value	Δ skew	Δ skew P-value	Δ kurtosis	Δ kurtosis P-value
Syn100	-0.001	.6299	0.000	.0652	1.063	.0825	3.317	.1914
ABIDE-I	0.000	.5195	0.000	.3342	0.167	.3996	0.151	.4853
ABIDE-II	-0.001	.6410	0.000	.2069	-0.061	.5740	-0.685	.7858
HCP-EP	0.001	.2646	0.000	.5192	-0.334	.8415	-0.771	.7579
BANDA	-0.001	.6802	0.000	.5319	-0.059	.5593	-0.396	.6361
DCAM	0.001	.2364	0.000	.0838	0.542	.0510	1.005	.1540
PDC	0.000	.4445	0.000	.4128	0.271	.2317	1.192	.1583

Table S5: Group differences in betweenness centrality distributions for positive-only SCNs. P-values were calculated via permutation tests with $n = 10,000$ permutations; $n = 62$ observations for all tests.

Sample	Δ mean	Δ mean P-value	Δ variance	Δ variance P-value	Δ skew	Δ skew P-value	Δ kurtosis	Δ kurtosis P-value
Syn100	0.001	.2850	0.000	**.0316**	0.673	.3195	2.560	.3951
ABIDE-I	0.001	.4029	0.000	.4606	-0.196	.7202	0.061	.4641
ABIDE-II	0.001	.3901	0.000	.1160	-0.214	.6565	-0.768	.6598
HCP-EP	-0.001	.5839	0.000	.9803	-1.186	.9301	-3.681	.7358
BANDA	0.000	.4554	0.000	.7184	-0.475	.7501	-1.881	.7226
DCAM	0.001	.4296	0.000	.1558	0.240	.3207	-0.552	.6088
PDC	0.002	.2565	0.000	.2186	0.340	.2867	1.772	.2665

Table S6: Group differences in betweenness centrality distributions for negative-only SCNs. P-values were calculated via permutation tests with $n = 10,000$ permutations; $n = 62$ observations for all tests.

Sample	FC network F(6, 3775)	FC network P-value	Visual t	Visual P-value	Somatomotor t	Somatomotor P-value	DA t	DA P-value	VA t	VA P-value	Limbic t	Limbic P-value	FP t	FP P-value	Default t	Default P-value
Syn100	3.97	**.0006**	-0.08	.9390	0.09	.9283	-0.12	.9083	0.51	.9116	0.51	.6110	0.15	.8811	-0.27	.7855
ABIDE-I	0.46	.8379	0.12	.9018	-0.03	.9777	0.01	.9937	-0.23	.8090	-0.23	.8192	0.18	.8566	-0.30	.7630
ABIDE-II	2.96	**.0070**	-0.05	.9589	-0.21	.8341	-0.01	.9926	-0.11	.7942	-0.11	.9086	-0.12	.9075	0.19	.8530
HCP-EP	3.19	**.0040**	0.19	.8478	0.13	.8974	0.14	.8894	0.13	.9064	0.13	.9002	-0.22	.8275	-0.18	.8547
BANDA	2.67	**.0137**	-0.05	.9618	-0.40	.6889	-0.08	.9330	0.01	.6950	0.01	.9903	0.10	.9170	0.04	.9667
DCAM	5.18	< **.0001**	-0.05	.9635	-0.09	.9257	0.05	.9583	-0.16	.9084	-0.16	.8709	0.03	.9728	0.05	.9636
PDC	1.44	.1952	-0.26	.7970	0.20	.8423	-0.30	.7655	0.33	.9666	0.33	.7444	-0.31	.7600	0.25	.8009

Table S7: Comparison of network weights across seven functional connectivity networks. Clusters were assigned to a network according to which network the majority of their parcels belonged. Each weight was assigned to two networks based on the consensus membership of its clusters. Mean group differences in weight (Z-scores) were compared across networks via one-way ANOVA, then the mean of each FC network was compared against that of all others via t-test. FC = functional connectivity, DA = dorsal attention, VA = ventral attention, FP = frontoparietal.

Sample	Group coefficient	Group coefficient SE	Group coefficient $t_{(3772.363)}$	Group coefficient P-value	Smooth term F	Smooth term P-value	Group:smooth deviance	Group:smooth P-value	n obs
Syn100	0.014	0.005	3.074	**.0021**	Control: 30.64<br>Aff: 30.06	Control: < **.0001**<br>Aff: < **.0001**	0.093	.0647	Control: 1009<br>Aff: 1014
ABIDE-I	0.006	0.005	1.268	.2051	Control: 74.02<br>Aff: 85.14	Control: < **.0001**<br>Aff: < **.0001**	0.051	.3616	Control: 958<br>Aff: 1005
ABIDE-II	0.012	0.005	2.507	**.0122**	Control: 92.03<br>Aff: 72.52	Control: < **.0001**<br>Aff: < **.0001**	0.055	.3170	Control: 985<br>Aff: 987
HCP-EP	-0.012	0.005	-2.457	**.0141**	Control: 57.86<br>Aff: 64.86	Control: < **.0001**<br>Aff: < **.0001**	0.035	.5593	Control: 986<br>Aff: 995
BANDA	0.007	0.004	1.606	.1085	Control: 26.91<br>Aff: 21.13	Control: < **.0001**<br>Aff: < **.0001**	0.040	.4001	Control: 1024<br>Aff: 997
DCAM	0.002	0.005	0.411	.6814	Control: 44.81<br>Aff: 59.08	Control: < **.0001**<br>Aff: < **.0001**	0.018	.8004	Control: 999<br>Aff: 986
PDC	-0.003	0.005	-0.604	.5461	Control: 19.97<br>Aff: 14.06	Control: < **.0001**<br>Aff: < **.0001**	0.028	.6264	Control: 980<br>Aff: 1003

Table S8: Generalised additive models (GAM) predicting positive network weights as a function of a parametric Group term, and separate smooth Distance terms for each group. Estimated and reference degrees of freedom for F-values can be found in Table S9.

Sample	Group coefficient	Group coefficient SE	Group coefficient $t_{(3772.363)}$	Group coefficient P-value	Smooth term F	Smooth term P-value	Group:smooth deviance	Group:smooth P-value	n obs
Syn100	-0.018	0.005	-4.013	< **.0001**	Control: 0.38<br>Aff: 0.59	Control: .5357<br>Aff: .4434	0.009	.3268	Control: 882<br>Aff: 877
ABIDE-I	-0.020	0.004	-4.411	< **.0001**	Control: 3.70<br>Aff: 8.60	Control: **.0192**<br>Aff: **.0034**	0.016	.2323	Control: 933<br>Aff: 886
ABIDE-II	-0.015	0.004	-3.361	**.0008**	Control: 3.77<br>Aff: 3.21	Control: **.0191**<br>Aff: **.0373**	-0.007	nan	Control: 906<br>Aff: 904
HCP-EP	0.011	0.005	2.301	**.0215**	Control: 4.15<br>Aff: 2.63	Control: **.0062**<br>Aff: **.0299**	-0.006	nan	Control: 905<br>Aff: 896
BANDA	0.000	0.004	-0.038	.9698	Control: 0.90<br>Aff: 0.43	Control: .3428<br>Aff: .5144	0.000	.8304	Control: 867<br>Aff: 894
DCAM	0.001	0.004	0.287	.7741	Control: 0.34<br>Aff: 1.68	Control: .5619<br>Aff: .1236	0.044	.0691	Control: 892<br>Aff: 905
PDC	-0.004	0.005	-0.912	.3621	Control: 2.62<br>Aff: 1.31	Control: .1060<br>Aff: .2000	0.026	.1445	Control: 911<br>Aff: 888

Table S9: Generalised additive models (GAM) predicting negative network weights as a function of a parametric Group term, a smooth Distance term and a Group:smooth Distance interaction. Estimated and reference degrees of freedom for F values can be found in Table S9.

Sample	F edf	F ref	F edf (pos)	F ref (pos)	F edf (neg)	F ref (neg)
Syn100	Control: 3.72<br>Aff: 3.92	Control: 3.96<br>Aff: 3.96	Control: 3.73<br>Aff: 3.93	Control: 3.96<br>Aff: 4.00	Control: 1.00<br>Aff: 1.00	Control: 1.00<br>Aff: 1.00
ABIDE-I	Control: 3.94<br>Aff: 3.95	Control: 4.00<br>Aff: 4.00	Control: 3.92<br>Aff: 3.92	Control: 4.00<br>Aff: 4.00	Control: 1.87<br>Aff: 1.00	Control: 2.32<br>Aff: 1.00
ABIDE-II	Control: 3.92<br>Aff: 3.94	Control: 4.00<br>Aff: 4.00	Control: 3.88<br>Aff: 3.93	Control: 4.00<br>Aff: 4.00	Control: 1.95<br>Aff: 1.69	Control: 2.42<br>Aff: 2.09
HCP-EP	Control: 3.93<br>Aff: 3.91	Control: 4.00<br>Aff: 4.00	Control: 3.94<br>Aff: 3.91	Control: 4.00<br>Aff: 4.00	Control: 2.48<br>Aff: 3.27	Control: 3.00<br>Aff: 3.72
BANDA	Control: 3.79<br>Aff: 3.85	Control: 3.97<br>Aff: 3.99	Control: 3.78<br>Aff: 3.68	Control: 3.97<br>Aff: 3.94	Control: 1.00<br>Aff: 1.00	Control: 1.00<br>Aff: 1.00
DCAM	Control: 3.91<br>Aff: 3.94	Control: 4.00<br>Aff: 4.00	Control: 3.87<br>Aff: 3.91	Control: 4.00<br>Aff: 3.91	Control: 1.00<br>Aff: 2.35	Control: 1.00<br>Aff: 2.86
PDC	Control: 3.84<br>Aff: 3.57	Control: 3.98<br>Aff: 3.89	Control: 3.81<br>Aff: 3.41	Control: 3.98<br>Aff: 3.81	Control: 1.00<br>Aff: 2.99	Control: 1.00<br>Aff: 3.50

Table S10: Degrees of freedom for F-tests across all GAMs. F-tests were performed for the significance of the smooth term of weight as a function of distance. ‘edf’ = estimated degrees of freedom, ‘ref’ = reference degrees of freedom.

Sample	SWP (positive)	delta (positive)	SWP (negative)	delta (negative)
Syn100	Control: 0.71<br>Aff: 0.71	Control: -1.00<br>Aff: -0.99	Control: 0.38<br>Aff: 0.32	Control: -0.51<br>Aff: -0.26
ABIDE-I	Control: 0.71<br>Aff: 0.71	Control: -1.00<br>Aff: -0.99	Control: 0.42<br>Aff: 0.35	Control: -0.73<br>Aff: -0.43
ABIDE-II	Control: 0.71<br>Aff: 0.71	Control: -0.98<br>Aff: -0.99	Control: 0.41<br>Aff: 0.36	Control: -0.70<br>Aff: -0.39
HCP-EP	Control: 0.71<br>Aff: 0.71	Control: -0.97<br>Aff: -0.98	Control: 0.33<br>Aff: 0.36	Control: -0.39<br>Aff: -0.46
BANDA	Control: 0.71<br>Aff: 0.71	Control: -0.98<br>Aff: -1.00	Control: 0.37<br>Aff: 0.38	Control: -0.48<br>Aff: -0.57
DCAM	Control: 0.71<br>Aff: 0.71	Control: -0.99<br>Aff: -1.00	Control: 0.34<br>Aff: 0.35	Control: -0.57<br>Aff: -0.35
PDC	Control: 0.71<br>Aff: 0.71	Control: -0.97<br>Aff: -1.00	Control: 0.33<br>Aff: 0.33	Control: -0.37<br>Aff: -0.35

Table S11: Small-world propensity (SWP) and delta values for positive- and negative-only SCNs.

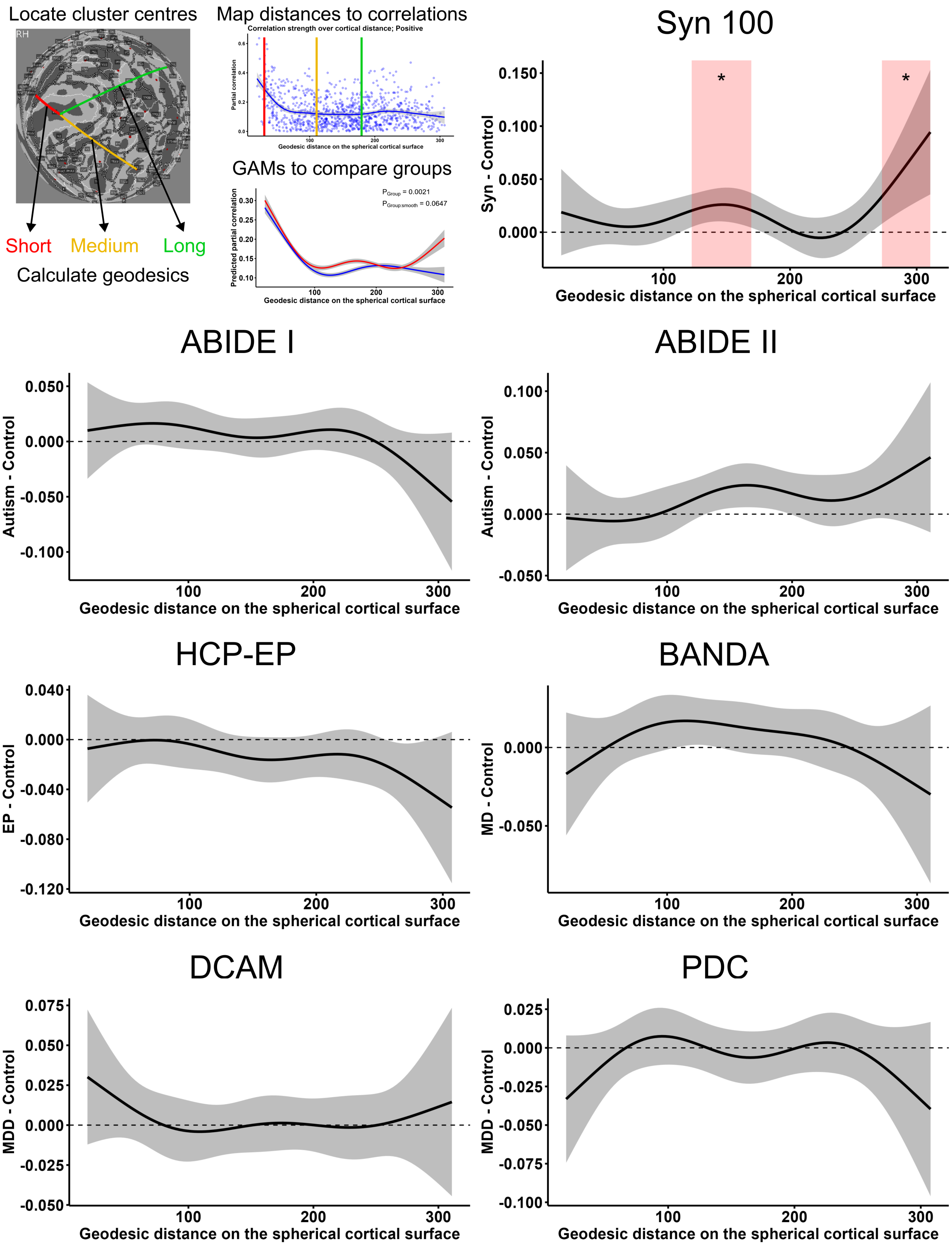

Figure S1: Group differences in positive-only network weights over the range of inter-cluster cortical distances. GAMs were used used to model the smooth relationship between cortical distance and network weight, according to group. The plots for each dataset show the group difference in predicted network weights across the range of cortical distances, with the shaded area indicating the 95% confidence interval. Distances with significant group differences are highlighted; '*' = $P < .05$, with FDR correction for 100 tests.

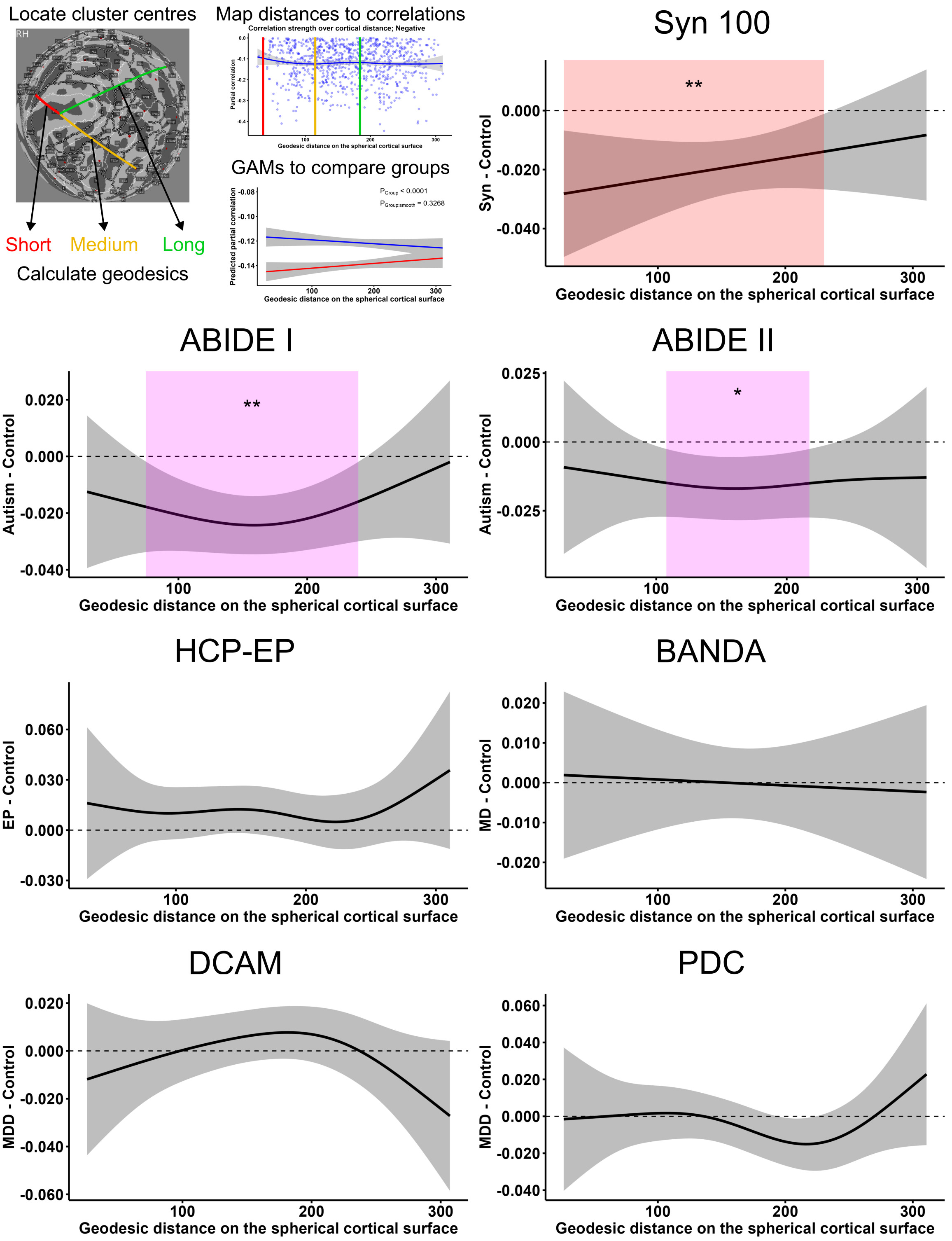

Figure S2: Group differences in negative-only network weights over the range of inter-cluster cortical distances. GAMs were used used to model the smooth relationship between cortical distance and network weight, according to group. The plots for each dataset show the group difference in predicted network weights across the range of cortical distances, with the shaded area indicating the 95% confidence interval. Distances with significant group differences are highlighted; '**' = $P < .01$, '*' = $P < .05$, with FDR correction for 100 tests. GAMs were fitted with $n = 1891$ observations per group.
